# Intravenous Foscarnet With Topical Cidofovir for Chronic Refractory Genital Herpes in a Patient With AIDS

**DOI:** 10.1177/2324709615621095

**Published:** 2015-12-11

**Authors:** Agnes Usoro, Alfreda Batts, Juan C. Sarria

**Affiliations:** 1 University of Texas Medical Branch School of Medicine, Galveston, TX, USA

**Keywords:** herpes, genital herpes, cidofovir, foscarnet, genital ulcer, wound care, hiv, aids

## Abstract

Few case reports have documented the use of topical cidofovir for refractory genital herpes simplex virus (HSV) ulcers in human immunodeficiency virus (HIV) infected patients. This drug formulation lacks a standardized concentration or even a procedural outline as to how it should be compounded. We aim to discuss the utilization of topical cidofovir in addition to presenting a procedural means of compounding it for treatment of refractory genital HSV ulcers. Our patient completed 21 days of intravenous foscarnet and 13 days of topical cidofovir with clinical improvement in the penile and scrotal ulcers. Genital herpes is a concern in patients with HIV because it generally manifests as a persistent infection. Physicians should be aware that when patients fail to respond to the conventional treatment regimens for genital HSV in a timely manner, other options are available, such as topical cidofovir as an adjuvant to systemic antivirals.

## Introduction

In the United States, 1 in 6 people ages 14 to 49 have genital herpes.^1^ Genital herpes is generally caused by *herpes simplex virus* (HSV) type 2, although rates of HSV type 1 infections are increasing, with the prevalence of genital herpes in patients with human immunodeficiency virus (HIV) infection approaching 50% to 90%.^2^ In immunocompetent individuals, mucocutaneous infections are generally self-limited. But in individuals with HIV, mucocutaneous infections are generally longer lasting with increased rates of recurrence and severity.^3^ The conventional treatment for genital HSV includes antiviral medications such as acyclovir, famiciclovir, and valacyclovir, which have similar efficacy in suppressing recurrent infections. The goal of therapy is to induce remission. Eradication of the virus is rare as HSV establishes itself as a chronic infection within the ganglion of sensory neurons during the primary infection.^2^ Intravenous acyclovir may be required for patients exhibiting severe or persistent infections but some may still experience poor clinical response. Here we report a case of chronic refractory genital HSV ulcers treated successfully with intravenous foscarnet and topical cidofovir. Written consent was obtained to document and submit for publication all information related to this case report.

## Case Report

**Figure 1.**
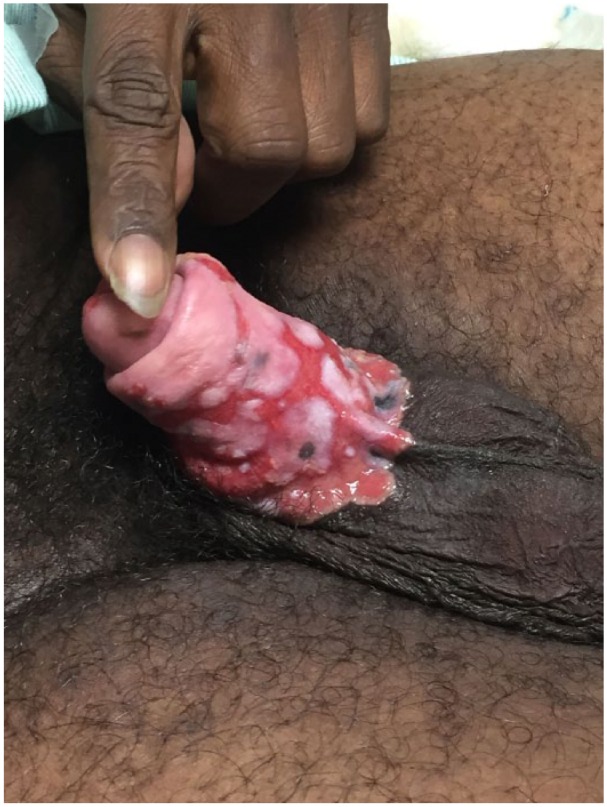
Penile ulcers at original presentation prior to initiation of intravenous foscarnet or topical cidofovir.

**Figure 2.**
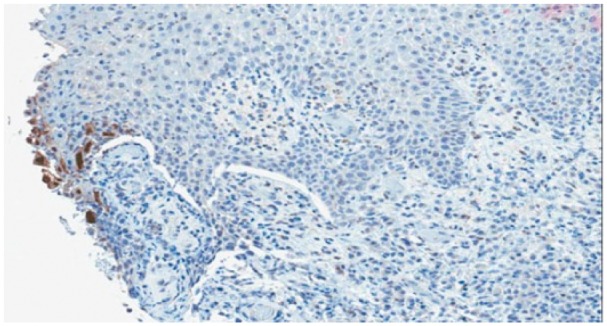
Immunohistochemistry staining (dark brown highlights) for HSV type 2.

A 48-year-old incarcerated male presented to the hospital with chronic penile and scrotal ulcers (Figure 1). He had a history of HIV infection (most recent CD4 count 143 cells/µL and HIV RNA viral load undetectable) and was recently switched to abacavir, ritonavir-boosted darunavir, and dolutegravir due to resistance to his previous antiretroviral regimen. The genital ulcers began a year prior to presentation on the distal glans of the penis as 2 small ulcers that progressed to involve the entire body of the penis and the left scrotum. He complained of penile tenderness but denied pain, fever, or constitutional symptoms. He had received previous treatment courses with oral acyclovir, a nucleoside analogue, and was taking 400 mg twice daily for chronic suppression. Diagnostic considerations included genital HSV, granuloma inguinale, and atypical syphilis. Intravenous foscarnet, another nucleoside analogue, 75 mg/kg twice daily, and oral doxycycline 100 mg twice daily were initiated. A biopsy of the ulcers was obtained. Tissue and swab cultures were negative for HSV but immunohistochemistry stain was positive for HSV-2 (Figure 2). Warthin-Starry stain was negative for Donovan bodies and spirochetes, and serum RPR was nonreactive. Granuloma inguinale and atypical syphilis were no longer considered, and doxycycline was discontinued after 4 days.

Complete blood counts and serum chemistries remained stable on treatment except for magnesium requiring daily replacement, allowing foscarnet to be titrated up to 90 mg/kg twice daily. Attempts were made to isolate HSV for resistance testing, but repeat tissue and swab cultures were negative. The genital ulcers were deemed to be secondary to HSV as evidenced by clinical response to foscarnet and positive immunohistochemistry staining despite negative viral cultures.

The penile ulcers began to show improvement after 7 days of foscarnet, but there still appeared to be areas of activity so additional treatment measures were considered. Literature review revealed several reports that outlined successful use of topical cidofovir and topical imiquimod for treatment of HSV cutaneous infections.^3-6^ Cidofovir is a nucleotide analogue that does not require enzyme activation for activity, unlike acyclovir and foscarnet. Imiquimod’s method of activity is not fully understood but believed to be related to stimulating the immune response through activation of toll-like receptors. Various concentrations of topical cidofovir have been cited, but a treatment algorithm for HSV genital ulcers recommends a 1% to 3% concentration.^7-9^ A request was made for the hospital pharmacy to compound topical cidofovir 1%, and while waiting for the pharmacy to approve and process the request, the patient was started on imiquimod 5% cream, which was commercially available and on the hospital formulary. On approval to compound topical cidofovir, the patient was transitioned to topical cidofovir 1%. The compounded formulation involved 375 mg of cidofovir in 37.5 g of an emollient base. Both imiquimod and cidofovir topical ointments were applied over the penile lesions with an overlying nonadherent Tefla pad, and secured with a Kerlix sterile gauze roll and tape. Dressing changes with topical medication was conducted by the patient, with nursing assistance, daily in the evening.

**Figure 3.**
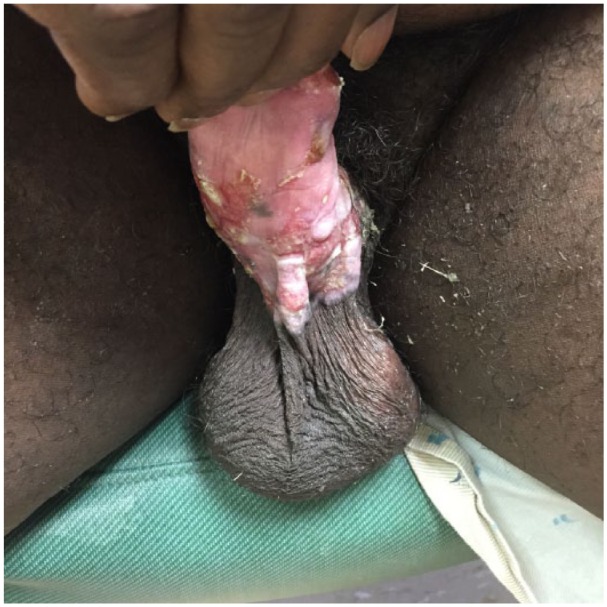
Penile ulcers after 21 days of intravenous foscarnet and 13 days of topical cidofovir.

The patient completed 21 days of intravenous foscarnet, 1 day of topical imiquimod 5%, and 13 days of topical cidofovir with significant clinical improvement in the penile and scrotal ulcers (Figure 3). Subsequently, the patient was transitioned to oral acyclovir 800 mg thrice daily for 1 month with plans of future transitioning to 400 mg twice daily. The rationale for utilizing oral acyclovir despite suspicions of an acyclovir-resistant infection came from studies revealing that isolated strains of HSV from a cutaneous ulcer exhibited reversion from acyclovir-resistant to acyclovir-susceptible following therapy with cidofovir.^5,7^ In addition, due to formulary constraints within the department of corrections, topical cidofovir was transitioned to topical imiquimod 5% thrice weekly for 1 month.

## Discussion

Immunocompromised persons who are infected with genital HSV tend to exhibit persistent and extensive ulcers, which commonly fail to respond to conventional antiviral regimens. The failed response is usually related to the infectious strain of HSV being resistant to conventional modalities such as acyclovir.^3^ It was assumed that our patient was infected with an acyclovir-resistant strain of HSV despite failed attempts to isolate the organism with viral tissue culture resulting in our inability to pursue resistance testing. This could be explained by an inadequate tissue biopsy sample or an inadequate preservation of the tissue specimen en route to the microbiology laboratory. Nonetheless, we approached the patient’s genital ulcers as acyclovir-resistant due to the chronicity of his infection despite suppressive acyclovir therapy.

Even though our patient received multiple concomitant treatments, significant clinical improvement was only observed after several days on combined intravenous foscarnet and topical cidofovir. Initial intravenous foscarnet monotherapy was only partially effective. Imiquimod was given briefly during induction treatment, and antiretroviral therapy did not lead to a robust CD4 recovery, indicating that a direct contribution of these treatments was less likely.

Genital herpes is a concern in patients with HIV infection because it generally manifests as a persistent infection, which, if not treated appropriately, can result in severe and disabling local disease. Physicians should be aware that when patients fail to respond to the conventional treatment regimens for genital HSV in a timely manner, other options are available. One such option is the use of topical cidofovir as an adjuvant to systemic antivirals such as foscarnet. Currently, topical cidofovir is not Food and Drug Administration approved for use in genital HSV infections, but with continued documentation of successful treatment responses, it is possible that this may become an additional indication. The majority of the documented responses are from case reports warranting the need for further studies.
